# LC-HRMS screening of per- and polyfluorinated alkyl substances (PFAS) in impregnated paper samples and contaminated soils

**DOI:** 10.1007/s00216-021-03463-9

**Published:** 2021-07-08

**Authors:** Boris Bugsel, Rebecca Bauer, Florian Herrmann, Martin E. Maier, Christian Zwiener

**Affiliations:** 1 Environmental Analytical Chemistry, Center for Applied Geoscience, University of Tübingen, Schnarrenbergstr. 94-96, 72076 Tübingen, Germany; 2 Institute for Organic Chemistry, University of Tübingen, Auf der Morgenstelle 18, 72076 Tübingen, Germany

**Keywords:** PFAS, HRMS, Soil, Paper, Perfluorinated carboxylic acids

## Abstract

High per- and polyfluorinated alkyl substance (PFAS) concentrations have been detected in agricultural soils in Southwest Germany. Discharges of PFAS-contaminated paper sludge and compost are suspected to be the cause of the contamination. Perfluorinated carboxylic acids (PFCAs) have been detected also in groundwater, drinking water, and plants in this area. Recently, previously unknown compounds have been identified by high-resolution mass spectrometry (HRMS). Major contaminants were polyfluorinated dialkylated phosphate esters (diPAPs) and N-ethyl perfluorooctane sulfonamide ethanol–based phosphate diester (diSAmPAP). In this study, HRMS screening for PFAS was applied to 14 soil samples from the contaminated area and 14 impregnated paper samples which were from a similar period than the contamination. The paper samples were characterized by diPAPs (from 4:2/6:2 to 12:2/12:2), fluorotelomer mercapto alkyl phosphates (FTMAPs; 6:2/6:2 to 10:2/10:2), and diSAmPAP. In soil samples, diPAPs and their transformation products (TPs) were the major contaminants, but also FTMAPs, diSAmPAP, and their TPs occurred. The distribution patterns of the carbon chain lengths of the precursor PFAS in soil samples were shown to resemble those in paper samples. This supports the hypothesis that paper sludge is a major source of contamination. The presence of major degradation products like PFCAs, FTSAs, or PFOS and their distribution of carbon chain lengths indicate the activity of biotic or abiotic degradation processes and selective leaching processes from the upper soil horizons.

**Graphical abstract:**

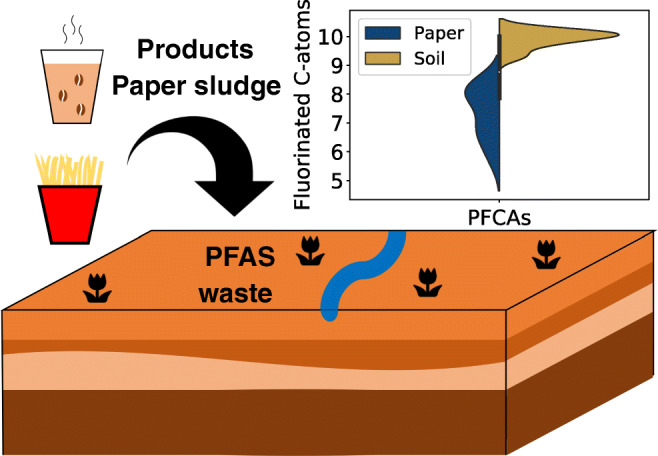

**Supplementary Information:**

The online version contains supplementary material available at 10.1007/s00216-021-03463-9.

## Introduction

Per- and polyfluorinated alkyl substances (PFAS) have unique properties and a broad application spectrum. Currently, PFAS comprise a family of approximately 4730 known individual compounds [1]. They are chemically and thermally very stable compounds [2] and characterized by hydrophobic and lipophobic properties. Therefore, PFAS are advantageous compounds in many consumer products and in industrial processes. They are widely used as oil and water repellents for textiles [3] and paper products [4], and also as surfactants in fire-fighting foams [5] and industrial processes like electroplating [6]. However, concerns about PFAS come from their high persistence and their potential to bioaccumulate and to cause adverse health effects in laboratory and wild animals [7–10]. Due to the widespread and extensive use of PFAS, they are found in aquatic and terrestrial fauna, in human blood serum [11], and in ground, surface, and drinking water [12–17].

Many commercially used PFAS have the potential to form transformation or dead-end products in the environment and are therefore referred to as precursor compounds. Typical PFAS precursors that were applied to food contact paper in the past include polyfluorinated dialkylated phosphate esters (diPAPs) along with their mono- and trialkylated analogues as impurities (mono and triPAPs) as well as N-ethyl perfluorooctane sulfonamide ethanol–based phosphate diester (diSAmPAP) [4].

Even though biotic and abiotic transformation can play a role for many precursor PFAS, perfluorocarboxylic acids (PFCAs) are typical dead-end transformation products (TPs) [18–21]. The use of PFAS in consumer and personal care products [22, 23] can lead to an input in the environment via different routes, like municipal wastewater treatment plants or landfill leachates [3].

In a recent case in SW Germany, the “Rastatt case,” high PFAS concentrations have been detected in soil, plants, groundwater, and even drinking water [24–27]. Deposition of compost mixed with waste from the paper industry onto arable land between the years 2000 and 2008 is suspected to be the main cause of contamination [28]. Our earlier study on the Rastatt site [24] focused on the determination and characterization of the contamination by liquid chromatography coupled to high-resolution mass spectrometry (LC-HRMS). It could be shown that diPAPs and diSAmPAP play a major role in the contamination of agricultural fields. Over time, diPAPs can degrade by cleavage of the phosphate ester bond which then results in a fluorotelomer alcohol (FTOH) [29]. Further degradation products which were detected in soil samples include n:2 fluorotelomer unsaturated carboxylic acids (FTUCAs), n:3 acids, and PFCAs [30]. This is in good agreement with a study by Liu and Liu (2016) who also found PFCAs as TPs from diPAP amended soils in aerobic degradation experiments [31]. Also diSAmPAP TPs were detected including N-ethyl perfluorooctane sulfonamidoacetate (EtFOSAA) and perfluorooctanesulfonate (PFOS) [32, 33].

To understand the big picture of the sources and fate of the contamination, we tested the hypothesis that the contaminant patterns on soils can be traced back to PFAS products used for paper impregnation. Therefore, in this work, we characterized PFAS and potential transformation products (TPs) from impregnated paper products from the early 2000s and compared the results to PFAS patterns from contaminated soil samples.

## Materials and methods

### Chemicals and reagents

Optima LC-MS grade methanol (MeOH), ammonium acetate (NH_4_Ac), and water were purchased from Fisher Scientific. Bis[2-(perfluorohexyl)ethyl]phosphate (6:2 diPAP), 6:2 fluorotelomer sulfonic acid (6:2 FTSA), N-ethyl polyfluoroalkyloctane sulfonamidoacetic acid (EtFOSAA), N-ethyl perfluorooctane sulfonamide ethanol–based phosphate diester (diSAmPAP), linear perfluorooctane sulfonic acid (PFOS), and perfluoroheptanoic acid (PFHpA) were purchased from Wellington Laboratories, Inc. (Guelph, Ontario, Canada). Perfluorooctanesulfonamide (FOSA) was obtained from SynQuest Laboratories (Alachua, FL, USA). 6:2 fluorotelomer mercapto alkyl phosphate (6:2 FTMAP) was synthesized according to literature [34]. Further details on the identification of the synthesized FTMAP by HRMS and NMR are given Supplementary Information (ESM1).

### Sample preparation

Homogenized, freeze-dried soil samples (S1–S14) from the plough horizon (top 30 cm) were provided by the Agricultural Technology Center Augustenberg (Karlsruhe, Germany). All samples were from agricultural soil plots from two regions of about 1 million m^2^ with known PFAS contamination in SW Germany [35]: Rastatt/Baden-Baden (S1–S10, S12) and Mannheim (S11, S13, S14). S1–S3 were sampled from one agricultural plot, S4–S7 from another plot about 8 km away. A blank sample S_B_ was taken from an agricultural test field of the Rastatt/Baden-Baden region where no intended input of PFAS occurred. Soil samples from deeper soil horizons were available from sites S1–S6, S9, S10 (30–60-cm depth) and S1–S6, S10 (60–90-cm depth) (for further details, see ESM1).

Five grams were weighed into a polypropylene (PP) tube and extracted with 10 mL methanol. The mixture was sonicated for 15 min and put onto a horizontal shaker for 24 h. The PP tube was subsequently centrifuged (15 min, 3000 rcf). An aliquot (1 mL) was transferred into a PP vial and centrifuged again prior to analysis (15 min, 6000 rcf).

Paper samples consisted of a variety of store-bought paper products including muffin liners (5 samples: P8, P9, P10, P12, P14) and paper plates (1 sample: P4) as well as raw paper materials (8 samples: P1, P2, P3, P5, P6, P7, P11, P13). Raw papers are plain paper products which are further used for the production of the finished consumer products. They were provided by the Fraunhofer Institute for Process Engineering and Packaging (Freising, Germany) and collected in the time period between 2000 and 2010. The papers were cut into rectangular shapes with defined surface areas in the range of 47–308 cm^2^ (details in Table S1, see ESM1), put in 5-L beakers, fully immersed in 100 mL MeOH, and shaken overnight. The beakers were tightly sealed with transparent foil to prevent evaporation of the MeOH. The extracts were then completely transferred into round-bottom flasks and reduced to < 5 mL with a rotary evaporator at 40 °C and 330 mbar. The enriched extracts were transferred into 5-mL glass vials and evaporated to 1 mL with a gentle stream of nitrogen. Prior to analysis, the samples were centrifuged (15 min, 6000 rcf) and transferred to a PP vial.

### Instrumental analysis

For soil samples S11–S14 and paper samples P1–P14, an Agilent C18 column (Poroshell 120 EC-C18, 2.1 mm × 100 mm, particle size 2.7 μm) with a flow rate of 0.4 mL/min and a temperature of 40 °C was used. Eluent A (95:5 v/v H_2_O/MeOH) and eluent B (95:5 v/v MeOH/H_2_O), both with 5 mM NH_4_Ac, were used for gradient elution. The gradient started with 25% B, followed by a linear increase to 85% B within 2 min and 100% B within 2.5 min. 100% B was kept isocratic until 12 min, followed by an equilibration time of the initial conditions until 15 min. And 20 μL were injected.

Soil samples S1–S10 were analyzed with a slightly different LC method. For soil samples S1–S10, an Acquity UPLC BEH C18 column (2.1 mm × 100 mm, particle size 1.7 μm) equipped with an Acquity UPLC BEH C18 guard column (2.1 mm × 5 mm, particle size 1.7 μm) with a flow rate of 0.4 mL/min and at a temperature of 60 °C was used. Eluent A (95:5 v/v H_2_O/MeOH) and eluent B (95:5 v/v MeOH/H_2_O), both with 2 mM NH_4_Ac, were used for gradient elution. The gradient started with 40% B, followed by a linear increase to 60% B within 1 min and 100% B within 1.5 min. 100% B was kept isocratic until 4.5 min, followed by an equilibration time of the initial conditions until 7 min. And 10 μL were injected.

All samples were measured on a 1290 HPLC system (Agilent Technologies, Waldbronn, Germany) coupled to a 6550 QTOF (Agilent Technologies, Santa Clara, USA) operated in negative ionization mode with a scan range from m/z 100 to m/z 1700.

### Data evaluation

Determination and identification of suspect compounds was determined according to the method described in our previous work [24]. Briefly, samples were screened for PFAS (a) by finding compounds in homologous series with CF2 repeating units and (b) by comparing the accurate masses of all features with exact masses in the OECD PFAS database. This database was manually extended with PFAS from literature research. Peak integration of confirmed compounds was performed using ProFinder 10.0 from Agilent Technologies. According to Schymanski et al. (2014) [36], we assigned confidence level 1a for compound identifications that were confirmed with an authentic standard; level 1b for homologues of an identified compound (1a) with systematic retention time shifts; and level 3 if only an accurate mass fit was available. No other levels were assigned (for further information on the systematic retention time shifts, see our previous publication (Bugsel & Zwiener (2020) [24])).

Python was used for statistical evaluation and plotting of the data, the heatmap was modified, and degradation schemes were drawn using InkScape. Boxplots were generated using the standard settings of the seaborn package in Python.

Due to different data acquisition methods from various sampling campaigns, the relative distribution patterns of homologues within a compound class for each sample have been compared. These patterns are generally independent of the concentrations and acquisition methods and can therefore be used across all samples. Commercial PFAS products typically contain homologue patterns from the industrial synthesis which may vary depending on manufacturer and batch, and are therefore useful as a sort of fingerprint.

For each compound class with multiple homologues, the weighted average perfluorinated carbon chain length for each sample *C*_*avg*_ can be calculated according to Eq. 1.

1 $$ {C}_{avg}=\sum \limits_{i=1}^k\left(\frac{A_i}{\sum {A}_i}\cdotp {n}_i\right) $$

where *A*_*i*_ is the peak area and *n*_*i*_ is the carbon chain length of the homologue *i*. For example, consider the detection of the three different PFCAs perfluoroheptanoic, -octanoic, and -nonanoic acid with *A*_*i*_= 0.7, 1, and 0.6, respectively. With ∑*A*_*i*_ = 2.3 and *n*_*i*_ = (6, 7, 8), the weighted average chain length would be *C*_*avg*_ = 6.96. Compound intensities within one homologous series are generally based on peak areas and not on concentrations, and may therefore differ from data based on actual concentrations. However, due to the non-target screening approach and the lack of analytical standards for most homologues, we used this approach, well-aware that ionization efficiency is known to vary even within a homologous series.

Since diPAPs and FTMAPs bear two fluorinated carbon chains, the calculated *C*_*avg*_ value was divided by two so that *C*_*avg*_ represents the fluorinated carbons for one chain only. This allows a better comparison of *C*_*avg*_ between precursor classes with two fluorinated carbon chains and TPs with one fluorinated carbon chain.

## Results and discussion

### Occurrence of PFAS classes

**Fig. 1:**
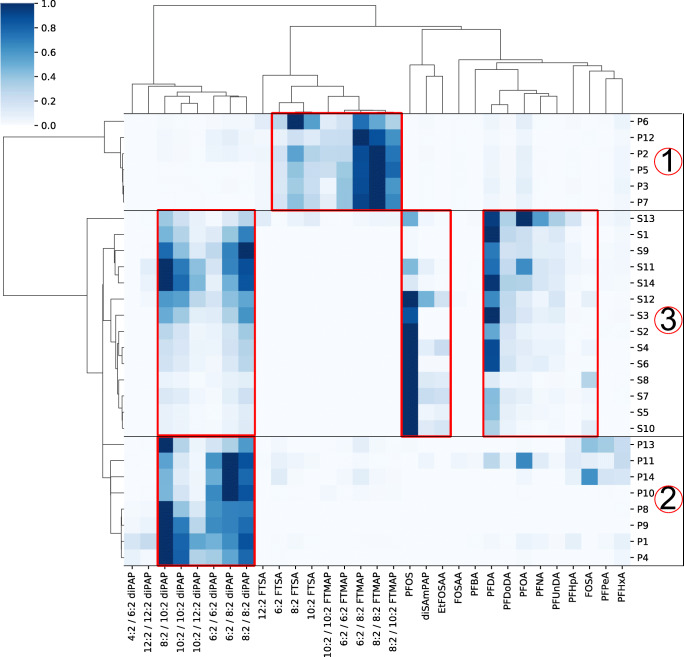
Clustermap for the occurrence and relative intensity of PFAS and potential TPs in paper (P1–P14) and soil (S1–S14) samples. Cluster 1, papers dominated by FTMAPs and TPs; cluster 2, papers with diPAPs; cluster 3, soil samples dominated by diPAPs, PFOS, and PFCAs. The normalized peak intensity for each sample is color coded. Dendrograms are generated using the correlation distance metric (ESM1)

PFAS-impregnated paper samples and PFAS-contaminated soil samples were analyzed by LC-HRMS to compare patterns of homologous series of PFAS. A total of 31 individual compounds out of 8 different compound classes were identified in all samples using a previously described method [24]. The method is briefly described in the “Data evaluation” section. The control sample showed low levels of PFOS; no other PFAS were found. The full result set including signal abundances, mass deviations, and confidence levels of identification in all paper samples, soil samples, and the control soil sample are given in ESM2. The compound classes PFCAs, diPAPs, diSAmPAP, EtFOSAA, FOSA, FTSAs, and FTMAPs were confirmed with one authentic standard for each homologous series. In soil samples S11 and S12, FOSAA was confirmed by its accurate mass with an error less than 3 ppm. Chemical structures of original contaminants, precursor compounds, and potential transformation products are shown in Figures S1, S2, and S3 (see ESM1). A clustermap visualizes the occurrence of different compound classes and their normalized abundances for all samples and allows to compare the chemical composition of the samples (Fig. 1). Clusters of samples and compounds are based on the correlation distance metric (for further information, see ESM1).

### Cluster analysis and relationship of compound classes

Interestingly, all samples are clearly subdivided into three clusters (Fig. 1): Cluster 1 consists of paper samples P2, P3, P5, P6, P7, and P12 which are dominated by FTMAPs and FTSAs; cluster 2 represents paper samples P1, P4, P8, P9, P10, P11, P13, and P14 which are dominated by diPAPs; and cluster 3 comprises soil samples S1–S14 which are mainly characterized by diPAPs, PFOS, and PFCAs.

In cluster 1, FTMAPs range from 6:2/6:2 to 10:2/10:2 and are typically found in commercial products for paper impregnation like Lodyne P208E (Trier et al. 2011). For example, from paper P2, 9 mg/m^2^ of 6:2/6:2 FTMAP were extractable by methanol (see ESM1 for further information). FTSAs range from 6:2 to 12:2 and are suggested to be processing aids or reagents of FTMAP synthesis.

In paper samples of cluster 2, diPAPs dominate in the range between 4:2/6:2 and 12:2/12:2. They are used in commercial fluorinated impregnation products like Zonyl [37].

All soil samples in cluster 3 are dominated by diPAPs (4:2/6:2 to 12:2/12:2), PFOS, and PFCAs. Especially perfluorodecanoic acid (PFDA) occurs in all soil samples (S1–S14). PFCAs are original contaminants in consumer products and potential break-down products of diPAPs [18, 30], which explains their simultaneous occurrence in soil samples. Since the contamination with paper sludge was more than 10 years ago and there is still a rather constant input of PFCAs to groundwater [28], it is reasonable to assume that mobile PFCAs from the original contamination have been leached out from top soils and further delivery to soils is from ongoing degradation processes of precursors which are prevailing in the top soil.

PFOS in soils can be from original contamination, but is also a potential degradation product for example of diSAmPAP [24, 32], which co-occurs in high abundances in soil samples (S4, S7, S8, S10–S14). Further TPs of diSAmPAP are EtFOSAA, FOSAA, and FOSA which were also found in samples with high PFOS contamination. The co-occurrence of precursors and degradation products hint to the presence of active degradation processes, even if the kinetics may be rather slow since the precursor compounds are still being found 10 years after the last discharge. Interestingly, samples S2–S8, S10, and S12 with high PFOS contamination show less diPAP abundance and vice versa (S1, S9, S11, S13, S14). This is a strong hint to different sources of paper sludge on these soil plots.

**Fig. 2:**
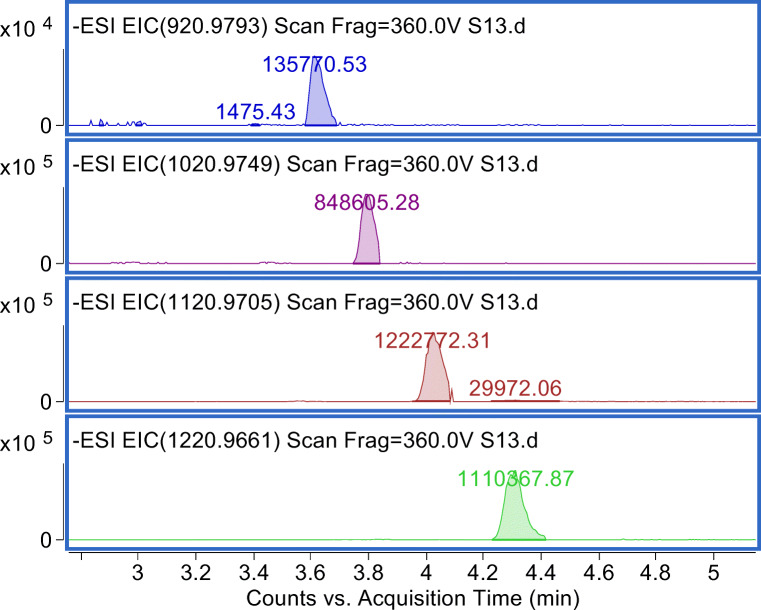
Extracted ion chromatograms for four FTMAPs in soil sample S13 (5-ppm window). 6:2/6:2 FTMAP (m/z 920.9812) was identified with the synthesized standard; further FTMAP homologues are characterized by the repeating unit C_2_F_4_ (Δ m/z 99.9936) and a systematic retention time shift

The soil sample S13 which appears in the clustermap at the upper fringe of cluster 3 (Fig. 1) is further characterized by the occurrence of five FTMAPs (6:2/6:2 to 10:2/10:2) and FTSAs (6:2 to 12:2) which hint to an additional contamination source compared to other soil samples. 8:2/10:2 FTMAP was further detected in S11 and S14 from the same region as S13. FTMAPs have been so far infrequently detected in the environment, e.g., 8:2 FTMAP in 1 out of 7 samples of landfill leachates below the LOQ (2 ng/L) [38]. Despite high production volumes and application to consumer products, the rare FTMAP detections may be due to faster degradation than other PFAS precursors and low mobility (hydrophobicity) [39]. The observation of FTMAPs in landfills with the input from consumer products supports the theory that paper sludge might have served as PFAS input. Figure 2 shows the extracted ion chromatograms of five FTMAP homologues and their characteristic retention time shifts. Their identity has been confirmed by accurate mass, accurate mass fragments, and retention time of the synthesized 6:2/6:2 FTMAP (ESM1). In addition to the occurrence of FTSAs as byproducts in paper, FTSAs may be break-down products of FTMAPs [34], which are formed by cleavage of the carbon-sulfur bond and further oxidation of the thiol group of the fluorotelomer side chain.

### Comparison of compound patterns and homologue distribution

**Fig. 3:**
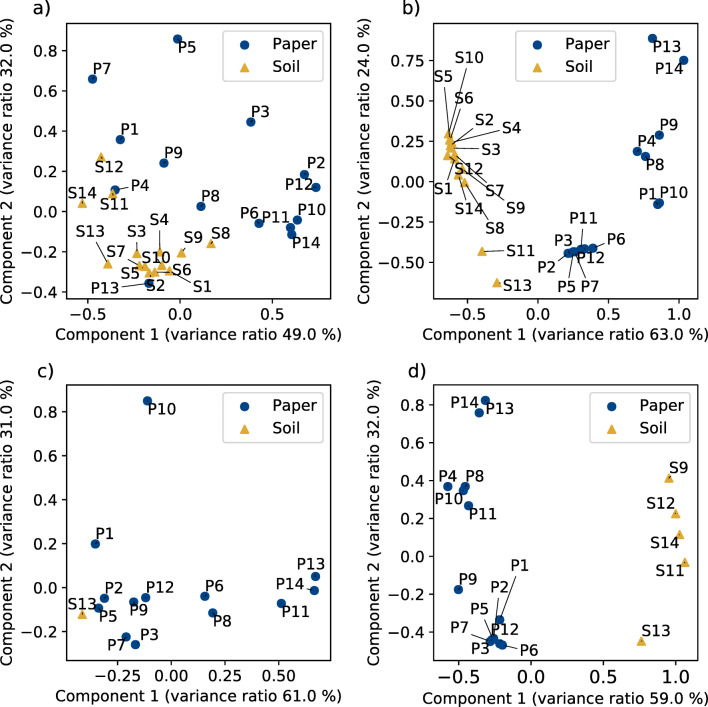
Principal component analysis (PCA) based on the normalized chain length distribution patterns in paper and soil samples for (**a**) diPAPs, (**b**) PFCAs, (**c**) FTMAPs, and (**d**) FTSAs

**Fig. 4:**
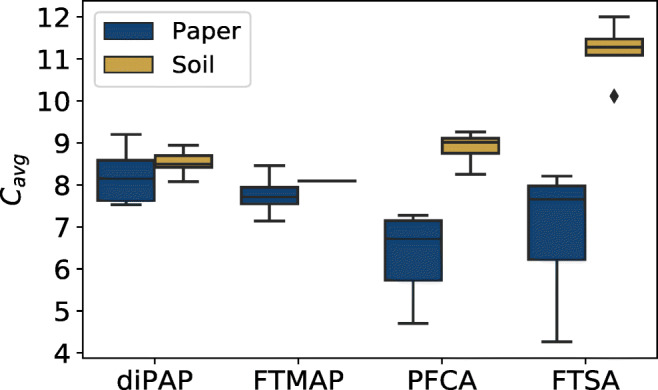
Box-whisker plot for the weighted average lengths of the perfluorinated carbon chain *C*_*avg*_ of diPAPs, FTMAPs, PFCAs, and FTSAs in 14 soil and 14 paper samples. For diPAPs and FTMAPs which bear two fluorotelomer carbon chains, only the average carbon length of one side chain was considered. The box represents 25 and 75 percentiles and the median, whiskers show the minimum and maximum values, and the diamond indicates an outlier

The results of the cluster analysis support the hypothesis that paper waste impregnated with PFAS precursors is the primary source of soil contamination which is reflected by PFAS patterns of impregnated paper products. Therefore, we compared the compound patterns in paper and soil samples in more detail and considered the distribution of homologues for the precursors diPAPs and FTMAPs, as well as for the transformation products PFCAs and FTSAs by principal component analysis (PCA) (Fig. 3) and boxplots demonstrating the average carbon chain lengths *C*_*avg*_ (Fig. 4). In the PCA plot for diPAPs in Fig. 3a, soil and paper samples show a rather broad distribution. DiPAPs in soils cluster in the lower left corner of the PCA plot and are partly separated from those in paper. Interestingly, the two paper samples P4 and P13 are well-positioned within the soil cluster, and are characterized by the abundant occurrence of 8:2/10:2 diPAP and rather low intensities of 6:2/6:2 and 6:2/8:2 diPAP. The results indicate a rather broad distribution of diPAP products used for paper impregnation and more specific, but still different input history and sources for soil contamination on the different soil plots which may be due to amount and frequency of sludge application and paper sludge from different production processes or manufacturers over time. Samples S1–S3 which are taken from one agricultural plot clustered very well. The same applies to S4–S7. It should still be emphasized here that the diPAP patterns in soil can also be changed by environmental processes. The PCA plots for PFCAs, the major products of biodegradation, show a different picture (Fig. 3b). A rather clear separation of paper vs. soil samples along component 1 reveals clear differences in the distribution pattern between the soil samples and the paper samples. Analogously, we see a similar picture for the PCA plots of FTMAPs (Fig. 3c), which shows a broad distribution for the paper samples but the occurrence in only one soil sample (S13). FTMAP patterns of S13 resemble well those of paper samples P2 and P5, which suggests again a rather specific source of contamination. The PCA plots of FTSAs, the major degradation products of FTMAPs, show again a clear separation between the soil and paper samples (Fig. 3d). The occurrence of FTSAs in five soil samples (S9, S11–14) further indicates a past FTMAP contamination of which only the degradation products are still visible. But also, direct FTSA discharge or contamination of so far unidentified precursors which may also form FTSAs has to be taken into consideration.

### Comparison of carbon chain lengths and environmental implications

Since various commercial PFAS products differ in their relative distribution of homologues, the differences between samples for individual compound classes can be worked out by the length of the carbon chains. We therefore plotted the weighted average length of the perfluorinated carbon chains *C*_*avg*_ for each sample in Fig. 4. For diPAPs, the distribution of *C*_*avg*_ in soil samples is narrower than that for paper samples, but both are still overlapping. The same applies to FTMAP which is only occurring in one soil sample. For both diPAPs and FTMAPs, we see a trend to longer *C*_*avg*_ in soil samples. For the TPs PFCAs and FTSAs, we clearly see higher *C*_*avg*_ values and therefore longer chain lengths in soil samples and a clear separation of the distribution patterns between paper and soil. The average carbon chain lengths of PFCAs in soil samples are at about 10 and for paper samples between 7 and 8, whereas for FTSAs, we found *C*_*avg*_ above 11 in soils and between 6 and 8 in papers. The data have to be interpreted against the background that the TPs can be introduced into soil via paper waste, but can also be formed in soil from the precursors diPAPs and FTMAPs by environmental processes. Therefore, we expect to find TPs with *C*_*avg*_ that fit to the original contamination of the so far unknown input material concerning TPs and PFAS precursors. The shift of PFCAs and FTSAs to longer chain lengths compared to their occurrence in paper and more importantly to their precursors diPAPs and FTMAPs can be explained by the fact that sorption and transport of PFAS precursors and TPs strongly depend on their carbon chain length.

Sorption of PFAS in soils and sediments is generally increased with perfluoroalkyl chain length [40–42]. Short-chain PFCAs and PFSAs with up to six carbon atoms showed early breakthrough in column leaching tests while their longer chain homologues were still retarded due to sorption to sediment [43]. This may explain the shift to long-chain PFCAs and FTSAs in the topsoil, whereas short-chain TPs have been already transported to deeper soil horizons by leachate. Since the soil-water distribution coefficients of the precursors diPAP and FTMAP which bear two polyfluorinated carbon chains are even higher than the corresponding PFCAs [42], they are expected to reside in the top soil within the plough horizon. This has been confirmed by the predominance of diPAPs in the upper 40 cm of a soil core [44] and by little or no detection in deeper soil layers from the same sites (n = 14/14 in 0–30-cm depth (S1–S14); n = 1/8 in 30–60-cm depth (S9); n = 0/7 in 60–90-cm depth; for further details, see Table S3 in ESM1; data of deeper soil layers not shown). Therefore, formation of PFCAs and FTSAs has occurred from corresponding precursors in the top soils, whereas short-chain PFCAs and FTSAs have been displaced by leachate to deeper soil horizons.

Overall, we can conclude that PFAS found in the soil samples are in good agreement with the PFAS used for paper impregnation. The paper impregnation products diPAPs, FTMAPs, and diSAmPAP and their TPs have been shown to play a major role in the soil contamination and their origin can be attributed to paper products. Differences in the patterns of TPs between paper and soil samples can be attributed mainly to sorption and leaching processes which are strongly dependent on carbon chain lengths.

## Supplementary Information

ESM 1 (PDF 835 kb)

ESM 2 (XLSX 36 kb)

## References

[1] OECD. Toward a new comprehensive global database of per- and polyfluoroalkyl subtances (PFASs) 2018. http://www.oecd.org/chemicalsafety/risk-management/global-database-of-per-and-polyfluoroalkyl-substances.xlsx. Accessed 04.06.2021.

[2] Kissa E (2001). Fluorinated surfactants and repellents: CRC Press.

[3] Lang JR, Allred BM, Peaslee GF, Field JA, Barlaz MA (2016). Release of per- and polyfluoroalkyl substances (PFASs) from carpet and clothing in model anaerobic landfill reactors. Environ Sci Technol.

[4] Trier X, Granby K, Christensen JH (2011). Polyfluorinated surfactants (PFS) in paper and board coatings for food packaging. Environ Sci Pollut R.

[5] Barzen-Hanson KA, Roberts SC, Choyke S, Oetjen K, McAlees A, Riddell N (2017). Discovery of 40 classes of per- and polyfluoroalkyl substances in historical aqueous film-forming foams (AFFFs) and AFFF-impacted groundwater. Environ Sci Technol.

[6] Glüge J, Scheringer M, Cousins I, DeWitt JC, Goldenman G, Herzke D (2020). An overview of the uses of per-and polyfluoroalkyl substances (PFAS). Environ Sci Process Impacts.

[7] Kim M, Son J, Park MS, Ji Y, Chae S, Jun C (2013). In vivo evaluation and comparison of developmental toxicity and teratogenicity of perfluoroalkyl compounds using Xenopus embryos. Chemosphere.

[8] Seacat AM, Thomford PJ, Hansen KJ, Clemen LA, Eldridge SR, Elcombe CR (2003). Sub-chronic dietary toxicity of potassium perfluorooctanesulfonate in rats. Toxicol.

[9] Dennis NM, Karnjanapiboonwong A, Subbiah S, Rewerts JN, Field JA, McCarthy C (2020). Chronic reproductive toxicity of perfluorooctane sulfonic acid and a simple mixture of perfluorooctane sulfonic acid and perfluorohexane sulfonic acid to northern bobwhite quail (Colinus virginianus). Environ Toxicol Chem.

[10] Barmentlo SH, Stel JM, van Doorn M, Eschauzier C, de Voogt P, Kraak MH (2015). Acute and chronic toxicity of short chained perfluoroalkyl substances to Daphnia magna. Environ Pollut.

[11] Olsen GW, Mair DC, Lange CC, Harrington LM, Church TR, Goldberg CL (2017). Per-and polyfluoroalkyl substances (PFAS) in American Red Cross adult blood donors, 2000–2015. Environ Res.

[12] McCord J, Strynar M (2019). Identification of per- and polyfluoroalkyl substances in the Cape Fear River by high resolution mass spectrometry and nontargeted screening. Environ Sci Technol.

[13] Yu N, Guo H, Yang J, Jin L, Wang X, Shi W (2018). Non-target and suspect screening of per- and polyfluoroalkyl substances in airborne particulate matter in China. Environ Sci Technol.

[14] Wang Y, Yu N, Zhu X, Guo H, Jiang J, Wang X (2018). Suspect and nontarget screening of per- and polyfluoroalkyl substances in wastewater from a fluorochemical manufacturing park. Environ Sci Technol.

[15] Pan Y, Zhang H, Cui Q, Sheng N, Yeung LWY, Sun Y (2018). Worldwide distribution of novel perfluoroether carboxylic and sulfonic acids in surface water. Environ Sci Technol.

[16] Chen H, Yao Y, Zhao Z, Wang Y, Wang Q, Ren C (2018). Multimedia distribution and transfer of per- and polyfluoroalkyl substances (PFASs) surrounding two fluorochemical manufacturing facilities in Fuxin. China Environ Sci Technol.

[17] Nguyen MA, Wiberg K, Ribeli E, Josefsson S, Futter M, Gustavsson J (2017). Spatial distribution and source tracing of per-and polyfluoroalkyl substances (PFASs) in surface water in Northern Europe. Environ Pollut.

[18] D'Eon J, Mabury S (2007). Production of perfluorinated carboxylic acids (PFCAs) from the biotransformation of polyfluoroalkyl phosphate surfactants (PAPs): exploring routes of human contamination. Environ Sci Technol.

[19] Mejia Avendano S, Liu J (2015). Production of PFOS from aerobic soil biotransformation of two perfluoroalkyl sulfonamide derivatives. Chemosphere..

[20] Wang N, Liu J, Buck RC, Korzeniowski SH, Wolstenholme BW, Folsom PW (2011). 6:2 fluorotelomer sulfonate aerobic biotransformation in activated sludge of waste water treatment plants. Chemosphere..

[21] Zabaleta I, Bizkarguenaga E, Izagirre U, Negreira N, Covaci A, Benskin JP (2017). Biotransformation of 8:2 polyfluoroalkyl phosphate diester in gilthead bream (Sparus aurata). Sci Total Environ.

[22] Fujii Y, Harada KH, Koizumi A (2013). Occurrence of perfluorinated carboxylic acids (PFCAs) in personal care products and compounding agents. Chemosphere..

[23] Ahrens L (2011). Polyfluoroalkyl compounds in the aquatic environment: a review of their occurrence and fate. J Environ Monit.

[24] Bugsel B, Zwiener C (2020). LC-MS screening of poly-and perfluoroalkyl substances in contaminated soil by Kendrick mass analysis. Anal Bioanal Chem.

[25] Muschket M, Keltsch N, Paschke H, Reemtsma T, Berger U. Determination of transformation products of per-and polyfluoroalkyl substances at trace levels in agricultural plants. J Chromagogr A. 2020;461271. 10.1016/j.chroma.2020.461271 32709323

[26] Biegel-Engler A, Vierke L, Apel P, Fetter E, Staude C (2017). Mitteilungen des Umweltbundesamtes zu per- und polyfluorierten Chemikalien (PFC) in Trinkwasser. Bundesgesundheitsbl.

[27] Janda J, Nödler K, Brauch H-J, Zwiener C, Lange FT (2019). Robust trace analysis of polar (C 2-C 8) perfluorinated carboxylic acids by liquid chromatography-tandem mass spectrometry: method development and application to surface water, groundwater and drinking water. Environ Sci Pollut R.

[28] Söhlmann R, Striegel G, Lange FT (2018). Die Anwendung der Summenparameter EOF und AOF bei der Untersuchung der Tiefenverlagerung von Perfluoralkyl- und Polyfluoralkylverbindungen (PFAS) in belasteten Böden in Mittelbaden. Mitt Umweltchem Ökotox.

[29] Lee H, D’eon J, Mabury SA (2010). Biodegradation of polyfluoroalkyl phosphates as a source of perfluorinated acids to the environment. Environ Sci Technol.

[30] Wang N, Szostek B, Buck RC, Folsom PW, Sulecki LM, Gannon JT (2009). 8-2 fluorotelomer alcohol aerobic soil biodegradation: pathways, metabolites, and metabolite yields. Chemosphere..

[31] Liu C, Liu J (2016). Aerobic biotransformation of polyfluoroalkyl phosphate esters (PAPs) in soil. Environ Pollut.

[32] Benskin JP, Ikonomou MG, Gobas FA, Begley TH, Woudneh MB, Cosgrove JR (2013). Biodegradation of N-ethyl perfluorooctane sulfonamido ethanol (EtFOSE) and EtFOSE-based phosphate diester (SAmPAP diester) in marine sediments. Environ Sci Technol.

[33] Zhang W, Pang S, Lin Z, Mishra S, Bhatt P, Chen S. Biotransformation of perfluoroalkyl acid precursors from various environmental systems: advances and perspectives. Environ Pollut. 2020;115908. 10.1016/j.envpol.2020.115908 33190976

[34] Lee H, Mabury SA (2011). A pilot survey of legacy and current commercial fluorinated chemicals in human sera from United States donors in 2009. Environ Sci Technol.

[35] Regierungspräsidium Karlsruhe. PFC-Problematik in Nord- und Mittelbaden: Statusbericht. Berichtszeitraum Januar bis Juni 2020. 2020. https://rp.baden-wuerttemberg.de/fileadmin/RP-Internet/Karlsruhe/Abteilung_5/Referat_54.1/Stabsstelle_PFC/_DocumentLibraries/Documents/Statusbericht_1._HJ_2020.pdf. Accessed 31.05.2021.

[36] Schymanski EL, Jeon J, Gulde R, Fenner K, Ruff M, Singer HP (2014). Identifying small molecules via high resolution mass spectrometry: communicating confidence. Environ Sci Technol.

[37] Trier X, Nielsen NJ, Christensen JH (2011). Structural isomers of polyfluorinated di- and tri-alkylated phosphate ester surfactants present in industrial blends and in microwave popcorn bags. Environ Sci Pollut R.

[38] Allred BM, Lang JR, Barlaz MA, Field JA (2014). Orthogonal zirconium diol/C18 liquid chromatography–tandem mass spectrometry analysis of poly and perfluoroalkyl substances in landfill leachate. J Chromagogr A.

[39] Wang Z, MacLeod M, Cousins IT, Scheringer M, Hungerbühler K (2011). Using COSMOtherm to predict physicochemical properties of poly-and perfluorinated alkyl substances (PFASs). Environ Chem.

[40] Enevoldsen R, Juhler RK (2010). Perfluorinated compounds (PFCs) in groundwater and aqueous soil extracts: using inline SPE-LC-MS/MS for screening and sorption characterisation of perfluorooctane sulphonate and related compounds. Anal Bioanal Chem.

[41] Higgins CP, Luthy RG (2006). Sorption of perfluorinated surfactants on sediments. Environ Sci Technol.

[42] Lee H, Mabury SA (2017). Sorption of perfluoroalkyl phosphonates and perfluoroalkyl phosphinates in soils. Environ Sci Technol.

[43] Vierke L, Möller A, Klitzke S (2014). Transport of perfluoroalkyl acids in a water-saturated sediment column investigated under near-natural conditions. Environ Pollut.

[44] Janda J, Nödler K, Scheurer M, Happel O, Nurenberg G, Zwiener C (2019). Closing the gap - inclusion of ultrashort-chain perfluoroalkyl carboxylic acids in the total oxidizable precursor (TOP) assay protocol. Environ Sci Process Impacts.

