# Supplementary material for: LC-HRMS screening of per- and polyfluorinated alkyl substances (PFAS) in impregnated paper samples and contaminated soils
Source: Anal Bioanal Chem. 2021 Jul 8;414(3):1217–25. doi: 10.1007/s00216-021-03463-9 (PMC8724098; doi:10.1007/s00216-021-03463-9)
Supplement: Supplementary file 1 — (PDF 835 kb) [file 216_2021_3463_MOESM1_ESM.pdf]

Supplementary Material

LC-HRMS screening of per- and polyfluorinated alkyl substances (PFAS) in impregnated paper samples and contaminated soils

Boris Bugsel[1], Rebecca Bauer[1], Florian Herrmann[2], Martin E. Maier[2], Christian Zwiener[1]

[1] Environmental Analytical Chemistry, Center for Applied Geoscience, University of Tübingen, Schnarrenbergstr. 94-96, 72076 Tübingen, Germany

[2] Institute for Organic Chemistry, University of Tübingen, Auf der Morgenstelle 18, 72076 Tübingen

Contents

Paper extraction 2

List of soil samples including deep soil layers 3

Clustermap calculation 3

Precursor classes, TP classes & suggested degradation schemes 4

Homologue patterns 6

Synthesis and identification of 6:2 FTMAP 6

PFAS identification by standards 11

LC-MS Operating parameters

Table S1: *Operating parameters used for HPLC-QTOF measurements*

Instrument Parameters	6550 QTOF	6470 QqQ
Gas Temp (°C)	150	150
Gas Flow (L/min)	16	16
Nebulizer (psig)	35	45
Sheath gas temperature (°C)	380	380
Sheath gas flow (L/min)	12	12
Capillary voltage (V)	3000	3000
Nozzle voltage (V)	300	0

Paper extraction

Table S2: *Paper size used for extraction.*

Sample ID	Size [cm^2]
P1	307
P2	307
P3	307
P4	151
P5	307
P6	307
P7	307
P8	104
P9	104
P10	82
P11	47
P12	74
P13	89
P14	79

List of soil samples including deep soil layers

Table S3: Samples that were part of this study are marked with x. Samples S1-S14 from the 0 cm – 30 cm horizon are discussed in the main text. Various diPAP homologues were found in all samples marked with an asterisk. n/a means that no sample from the sampling site at the respective depth was available.

Sampling site	0 cm – 30 cm	30 cm – 60 cm	60 cm – 90 cm
S1	x*	x	x
S2	x*	x	x
S3	x*	x	x
S4	x*	x	x
S5	x*	x	x
S6	x*	x	x
S7	x*	n/a	n/a
S8	x*	n/a	n/a
S9	x*	x*	n/a
S10	x*	x	x
S11	x*	n/a	n/a
S12	x*	n/a	n/a
S13	x*	n/a	n/a
S14	x*	n/a	n/a

Clustermap calculation

For the clustermap, distances between the samples were calculated according to the correlation distance metric which computes the correlation distance between two vectors u and v according to

$$1 - \frac{(u - \bar{u}) \cdot (v - \bar{v})}{||(u - \bar{u})||_2 ||(v - \bar{v})||_2}$$

where $||(x)||_p$ is the p-norm of x:

$$||(x)||_p \coloneqq \left(\sum_{i=1}^{n} |x_i|^p \right)^{\frac{1}{p}}$$

Precursor classes, TP classes & suggested degradation schemes

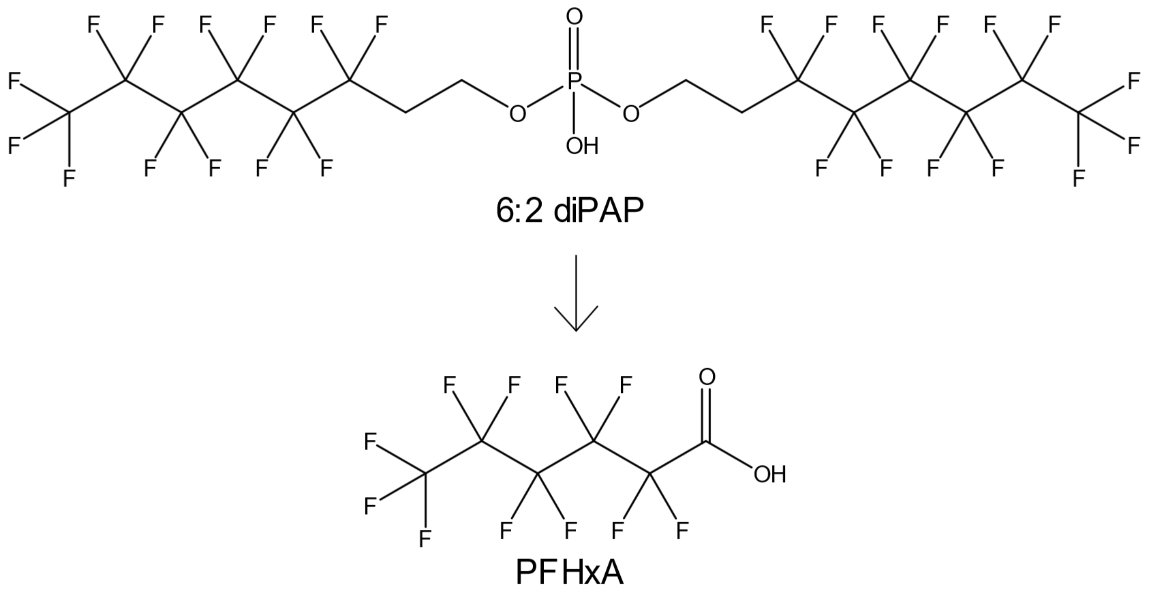

Figure S1: *6:2/6:2 diPAP and the major TP perfluorohexanoic acid (PFHxA).*

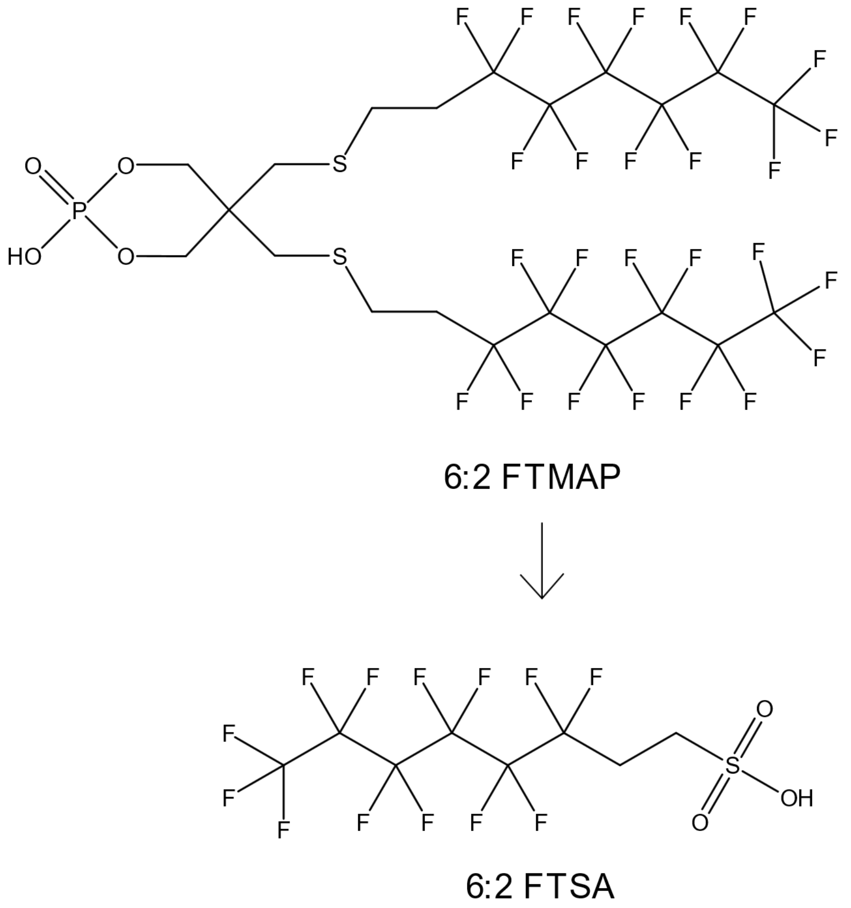

Figure S2: *6:2/6:2 FTMAP and the TP 6:2 FTSA.*

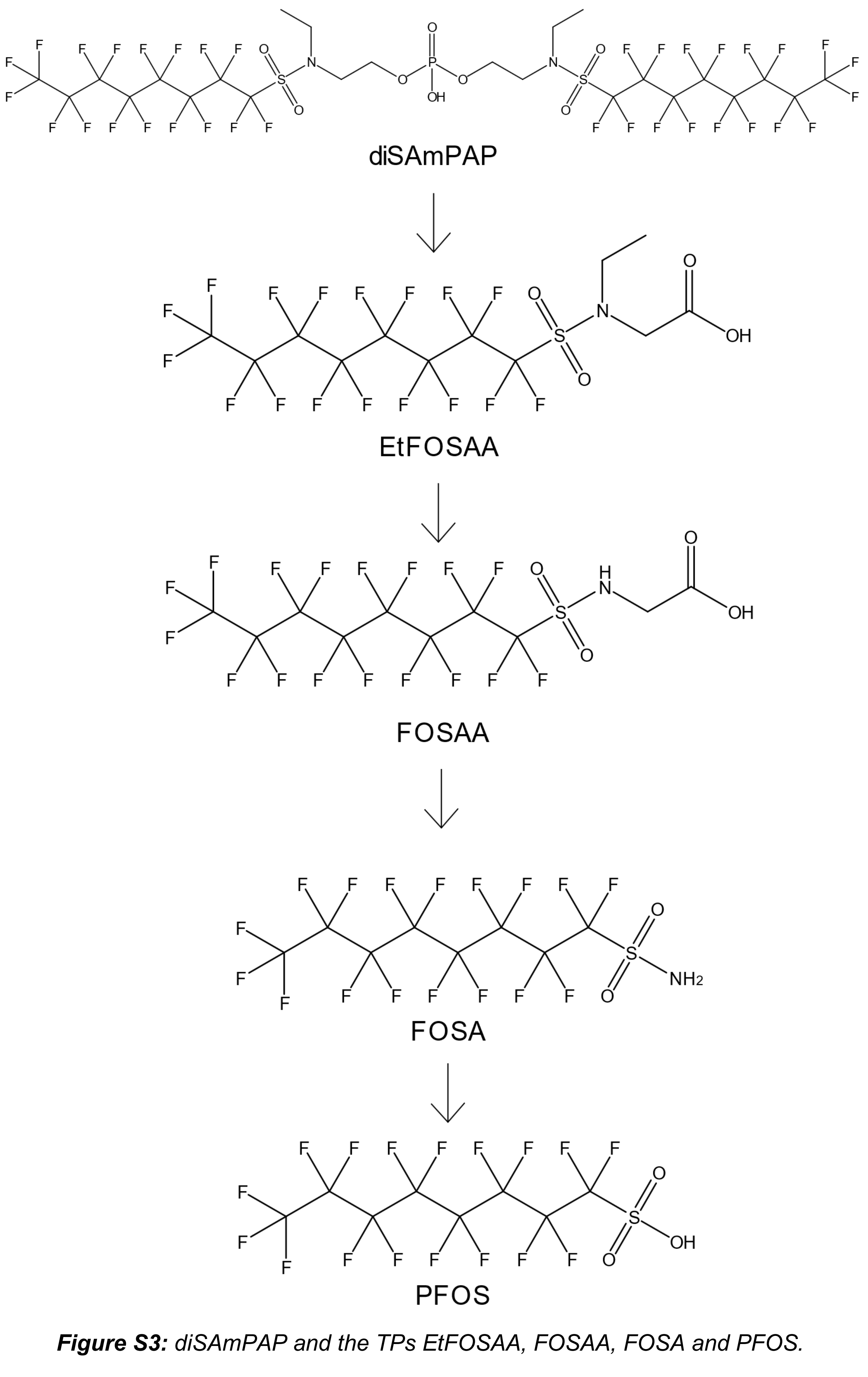

Figure S3: *diSAmPAP and the TPs EtFOSAA, FOSAA, FOSA and PFOS.*

Homologue patterns

Figure S4 shows the homologue pattern for diPAPs in soil sample S9. Relative signal intensities A are normalized by the signal intensity of 8:2/8:2 diPAP (A_{max}).

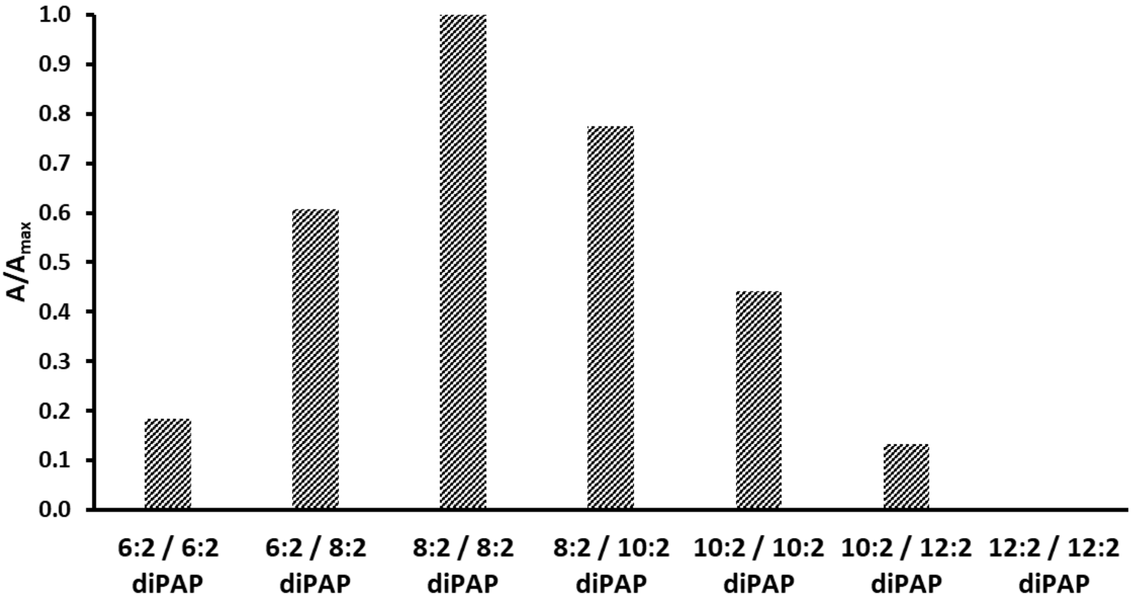

***Figure S4:** diPAP pattern in soil sample S9.*

Synthesis and identification of 6:2 FTMAP

6:2 FTMAP was synthesized according to the method supplied by Lee & Mabury (2011) [1]. Briefly, in the first step bis-(1H,1H,2H,2H-perfluorooctanethiolmethyl)-1,3-propanediol is obtained from a nucleophilic reaction of 1H,1H,2H,2H-perfluorooctanethiol with dibromopentyl glycol; the second reaction of bis-(1H,1H,2H,2H-perfluorooctanethiolmethyl)-1,3-propanediol with $POCl_3$ yields the final white solid product which was recrystallized with m-xylene. The product was identified by ^{1}H, ^{13}C, ^{31}P and ^{19}F NMR and by high-resolution MS (targeted MS/MS, CE = 40 eV; 6550 QTOF-MS from Agilent Technologies).

HRMS (spectrum in Fig. S5):

m/z 920.9813	$[M\text{-}H]^-$ (Δm 0.54 ppm)
m/z 880.9689	$[M\text{-}H\text{-}2HF]^-$ (Δm 0.5 ppm)
m/z 574.9785	$[C_{13}H_{13}F_{13}O_4PS_2]^-$ (Δm 1 ppm)
m/z 78.9590	$[PO_3]^-$ (Δm 0.7 ppm)

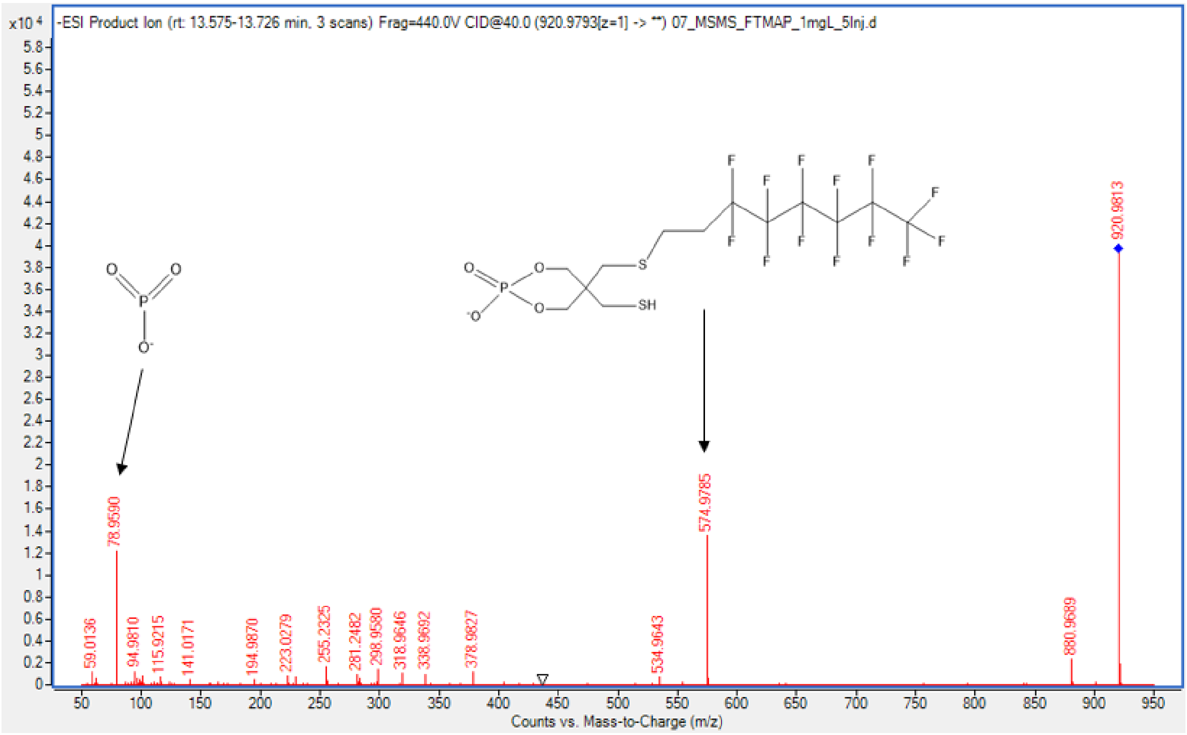

***Figure S5**: High resolution MS/MS fragmentation of the synthesized 6:2 FTMAP standard (1 mg/L; CE @ 40 eV).*

NMR

^{1}H (CD_3OD, 400 MHz)

δ = 2.46 – 2.57 (m, 4H, CH2)

δ = 2.69 (s, 4H, CH2)

δ = 2.8 – 2.88 (m, 4H, CH2)

δ = 3.53 (s, 4H, CH2)

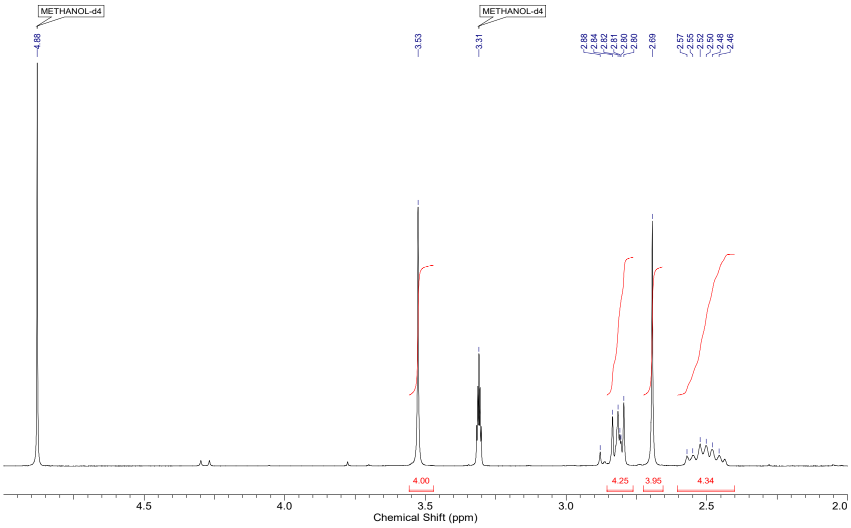

***Figure S6**: 1H NMR spectrum of the synthesized 6:2 FTMAP standard (in CD3OD, 400 MHz).*

^{19}F (CD_3OD, 377 MHz)

δ = 82.5 (CF3)

δ = 115.3-115.4 (m, CF2)

δ = 123.0 (s, CF2)

δ = 124.0 (s, CF2)

δ = 124.4-124.5 (d, CF2)

δ = 127.3-127.4 (m, CF2)

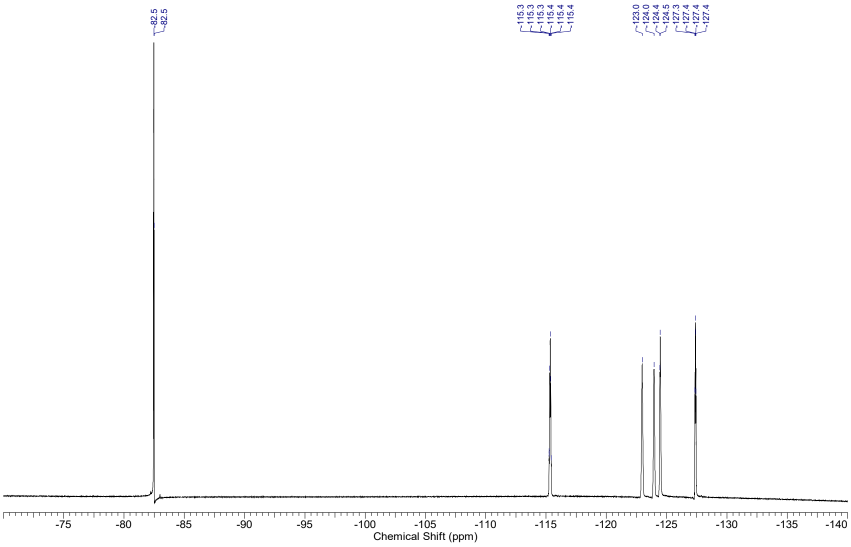

Figure S7: *19F NMR spectrum of the synthesized 6:2 FTMAP standard (in CD3OD, 377 MHz).*

^{13}C (CD_3OD, 100 MHz)

δ = 25.17
δ = 33.15
δ = 33.36
δ = 35.49
δ = 46.50
δ = 63.88

^{31}P (CD_3OD, 162 MHz)

δ = - 5.43

Identification of 6:2 FTMAP in paper and soil

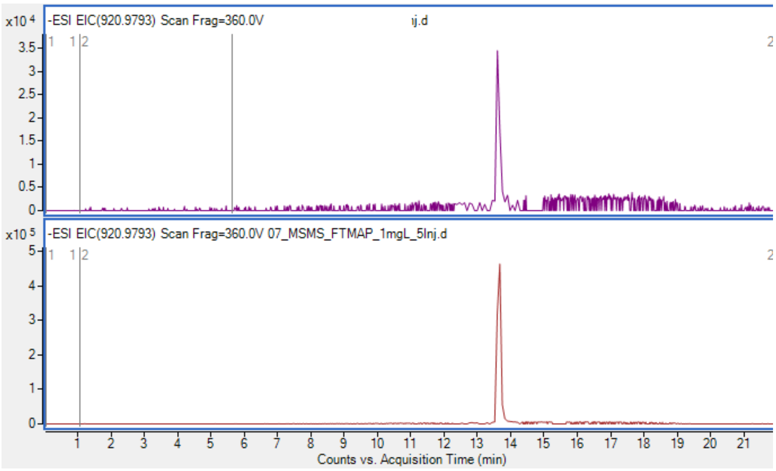

Figure S8: *Extracted ion chromatogram of m/z 920.9793 (10 ppm) in soil sample S13 (top) and the synthesized 6:2 FTMAP standard (bottom)*

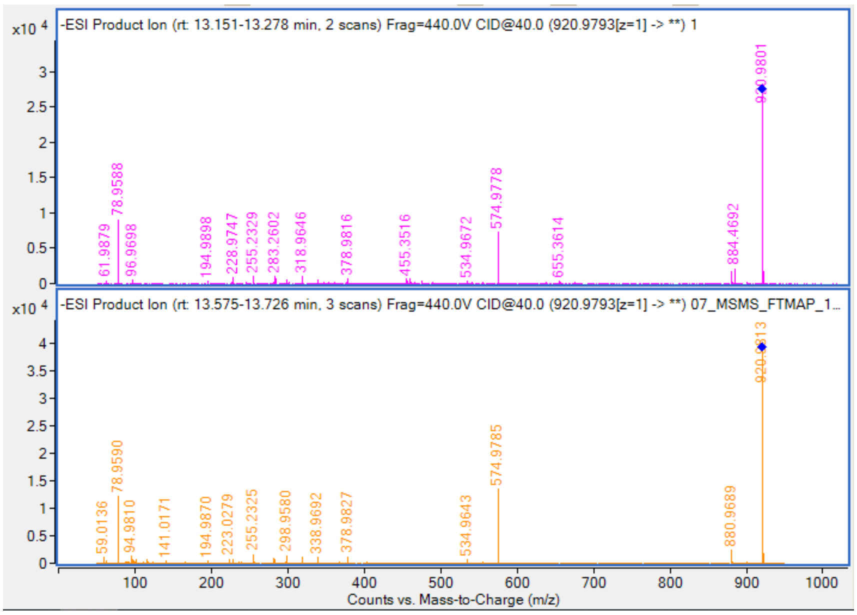

Figure S9*: MS/MS spectrum of 6:2 / 6:2 FTMAP in soil sample S13 (top) and a 1 mg/L standard solution, both @ 40 eV.*

Quantification of 6:2 FTMAP

6:2 FTMAP was quantified using a 1290 HPLC (Agilent Technologies, Waldbronn, Germany) coupled to a 6470 QqQ instrument. Eluent A (95:5 v/v H_2O/MeOH) and eluent B (95:5 v/v MeOH/H_2O), both with 2 mM NH_4Ac, were used for gradient elution. Mass transitions were measured in multiple reaction monitoring (m/z 921 -> m/z 575 @ 45 eV; m/z 921 -> m/z 79 @ 45 eV). A 6-point calibration curve ranging from 10 µg/L up to 200 µg/L (R^2 = 0.99) was used to estimate the concentration in sample P2 to 9.2 mg / m^2.

PFAS identification by standards

For mass deviations, see ESM2.xls.

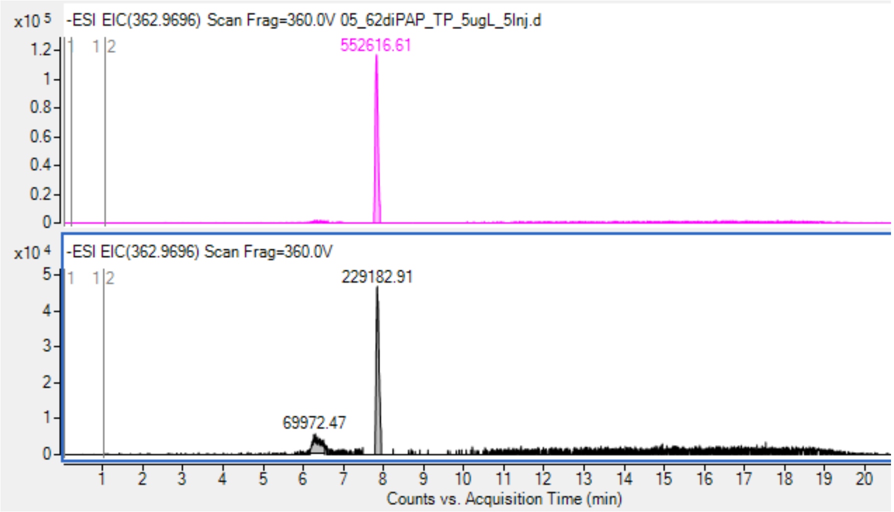

Figure S10: *PFHpA in a standard solution (top, 5 µg/L) and in soil sample S12 (bottom).*

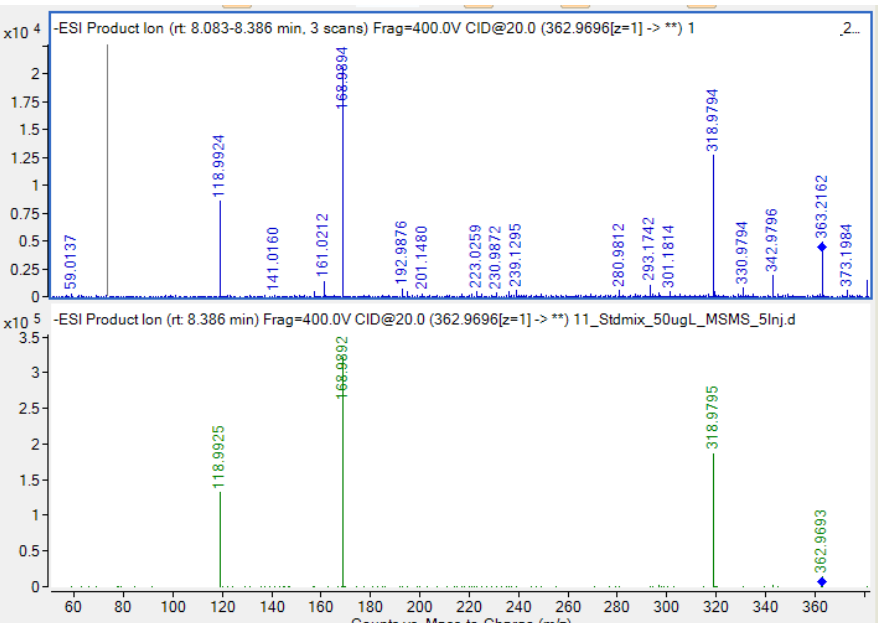

Figure S11*: MS/MS spectrum of PFHpA in soil sample S12 (top) and a 50 µg/L standard solution (bottom), both @ 20 eV.*

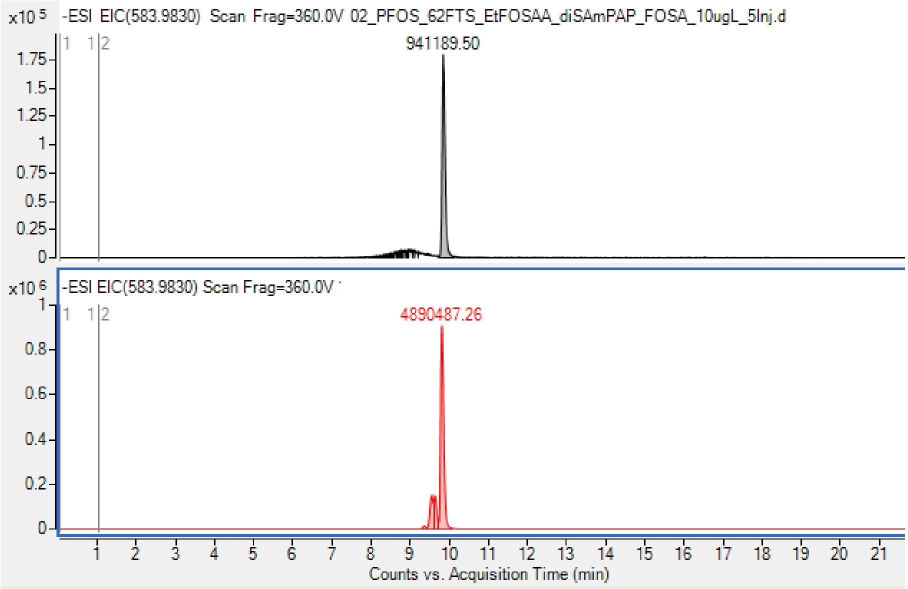

Figure S12: *EtFOSAA in a standard solution (top, 10 µg/L) and in soil sample S12 (bottom).*

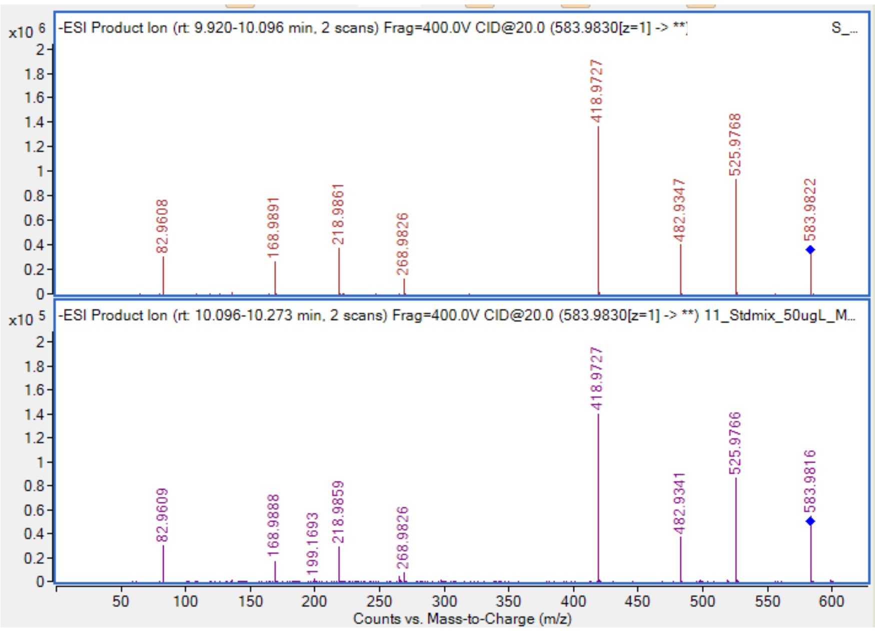

Figure S13*: MS/MS spectrum of EtFOSAA in soil sample S13 (top) and a 50 µg/L standard solution (bottom), both @ 20 eV.*

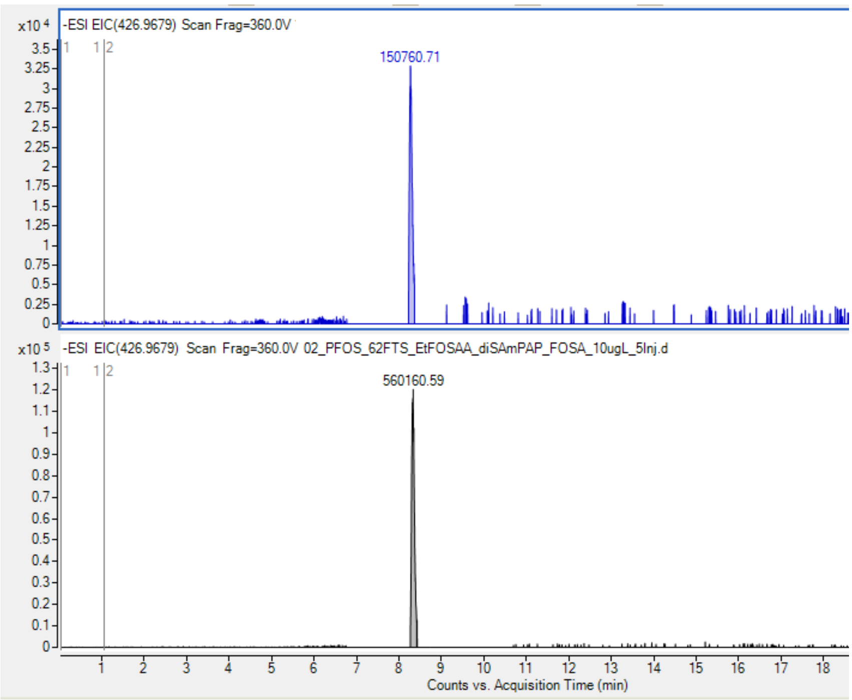

***Figure S14:** 6:2 FTSA in a standard solution (bottom, 10 µg/L) and in soil sample S13 (top).*

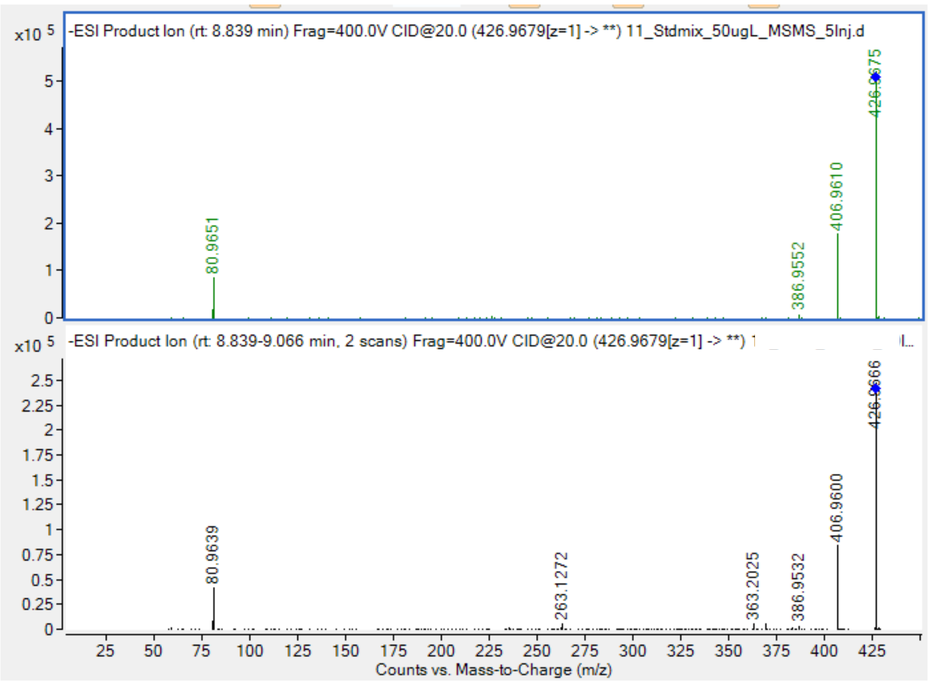

***Figure S15**: MS/MS spectrum of 6:2 FTSA in soil sample S13 (bottom) and a 50 µg/L standard solution (top), both @ 20 eV.*

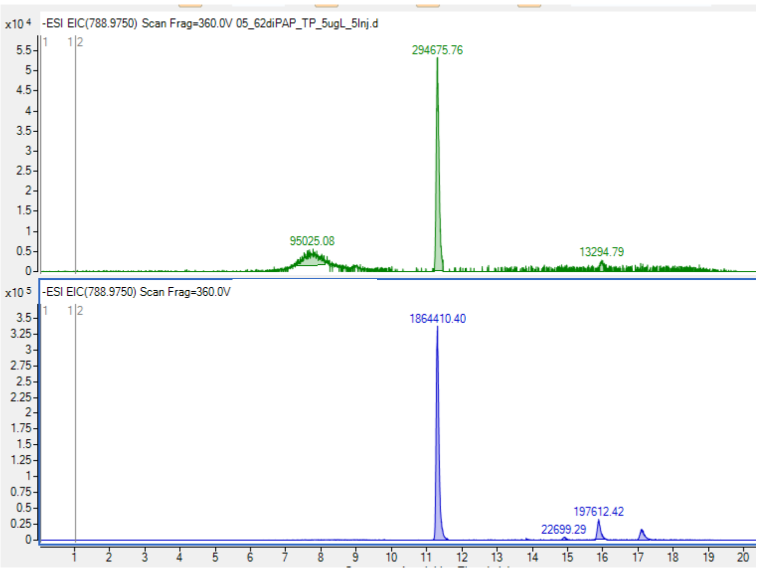

Figure S16: *6:2 diPAP in a standard solution (top, 5 µg/L) and in soil sample S12 (bottom).*

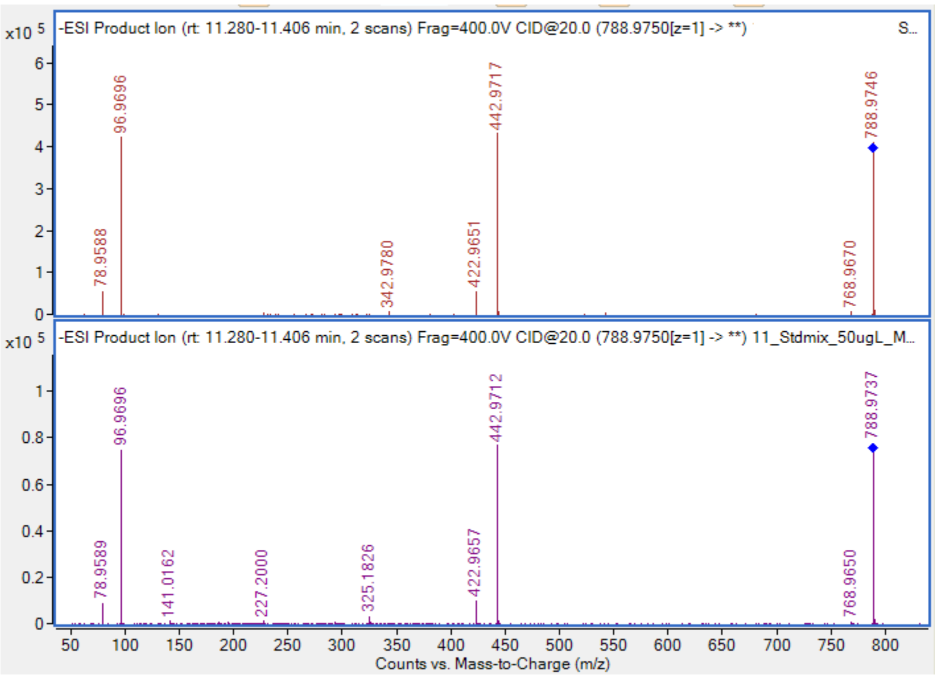

Figure S17*: MS/MS spectrum of 6:2 / 6:2 diPAP in soil sample S13 (top) and a 50 µg/L standard solution (bottom), both @ 20 eV.*

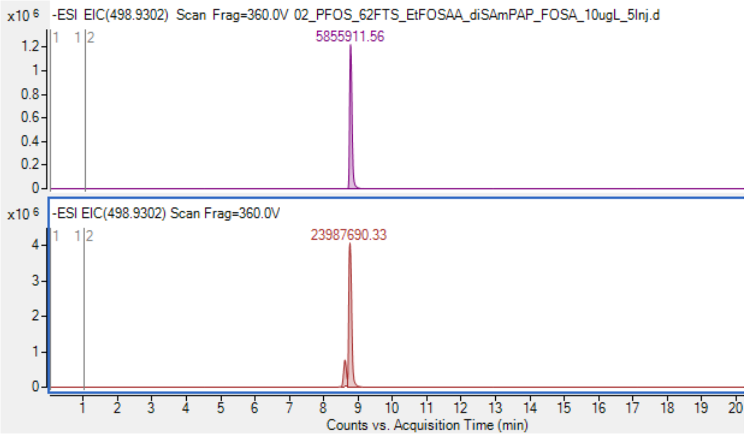

Figure S18: *PFOS in a standard solution (top, 10 µg/L) and in soil sample S12 (bottom). The double peak in soil sample S12 indicates branched isomers.*

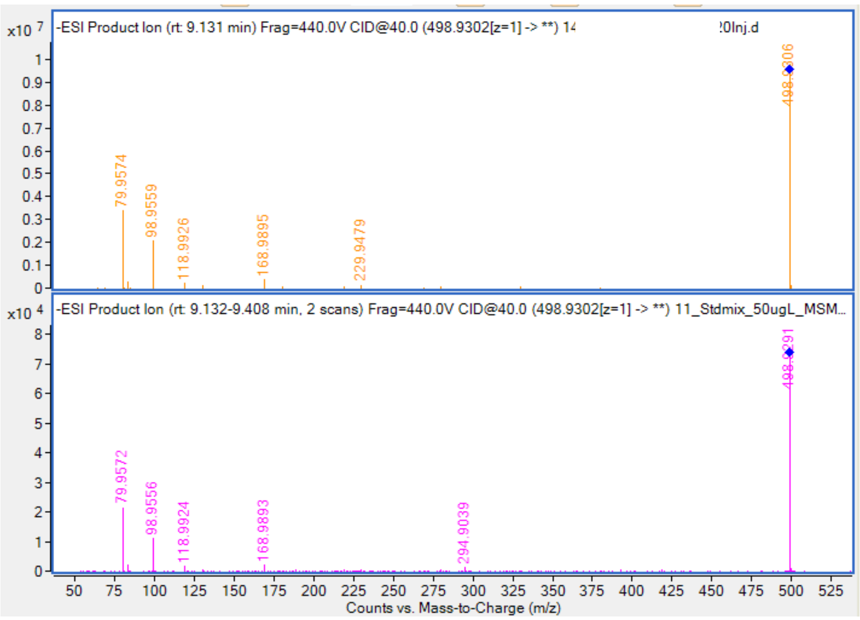

Figure S19: *MS/MS spectrum of PFOS in soil sample S13 (top) and a 50 µg/L standard solution, both @ 20 eV.*

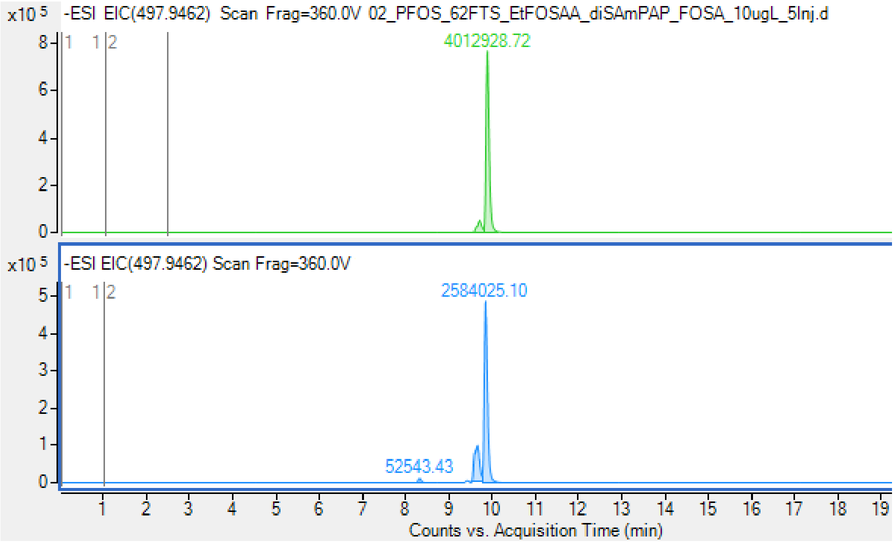

Figure S20: *FOSA in a standard solution (top, 10 µg/L) and in soil sample S12 (bottom). The double peak in soil sample S12 indicates branched isomers.*

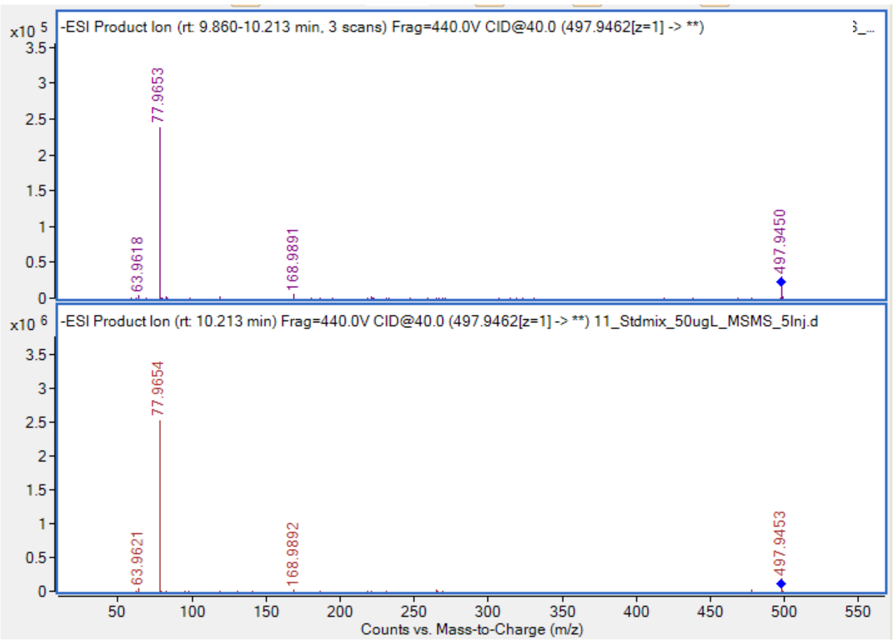

Figure S21*: MS/MS spectrum of FOSA in soil sample S12 (top) and a 50 µg/L standard solution (bottom), both @ 40 eV.*

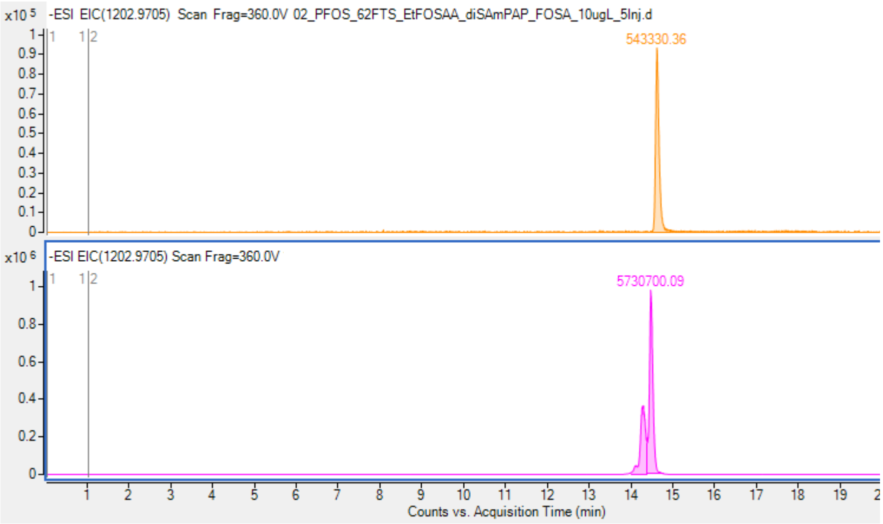

Figure S22: *diSAmPAP in a standard solution (top, 10 µg/L) and in soil sample S12 (bottom). The double peak in soil sample S12 indicates branched isomers.*

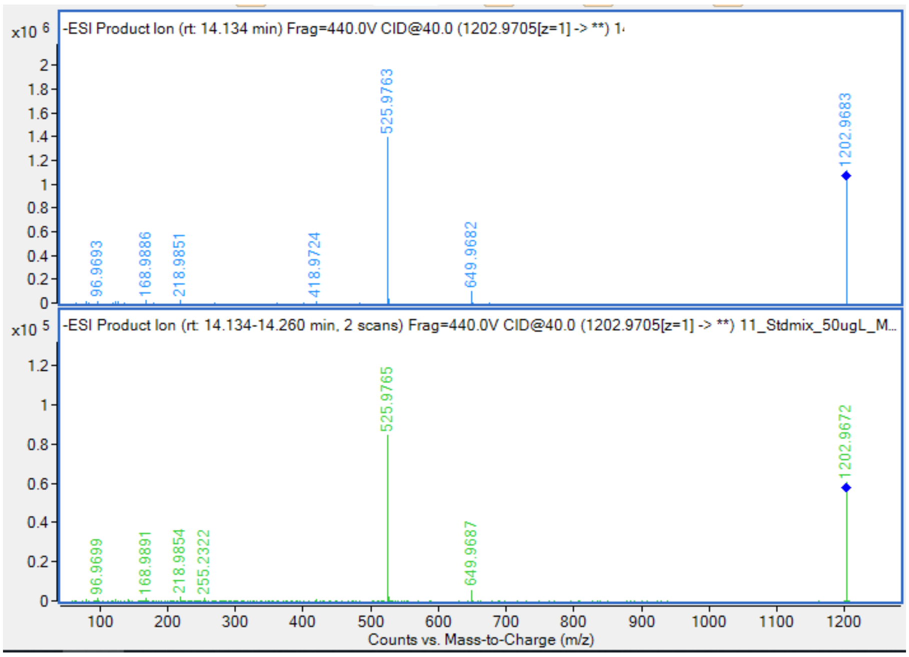

Figure S23: *MS/MS spectrum of diSAmPAP in soil sample S12 (top) and a 50 µg/L standard solution (bottom), both @ 40 eV.*

References

1. Lee, H.; Mabury, S. A., A pilot survey of legacy and current commercial fluorinated chemicals in human sera from United States donors in 2009. *Environmental Science & Technology* **2011,** *45*, (19), 8067-74.
